# ADSTGCN: A Dynamic Adaptive Deeper Spatio-Temporal Graph Convolutional Network for Multi-Step Traffic Forecasting

**DOI:** 10.3390/s23156950

**Published:** 2023-08-04

**Authors:** Zhengyan Cui, Junjun Zhang, Giseop Noh, Hyun Jun Park

**Affiliations:** 1 Department of Computer Information Engineering, Cheongju University, Cheongju 28503, Republic of Korea; cuizy1017@gmail.com (Z.C.); zjj416320@gmail.com (J.Z.); 2 Department of Artificial Intelligence Software, Cheongju University, Cheongju 28503, Republic of Korea; kafa46@cju.ac.kr

**Keywords:** traffic forecasting, spatio-temporal graph, deep graph convolutional network, adaptive graph construction

## Abstract

Multi-step traffic forecasting has always been extremely challenging due to constantly changing traffic conditions. Advanced Graph Convolutional Networks (GCNs) are widely used to extract spatial information from traffic networks. Existing GCNs for traffic forecasting are usually shallow networks that only aggregate two- or three-order node neighbor information. Because of aggregating deeper neighborhood information, an over-smoothing phenomenon occurs, thus leading to the degradation of model forecast performance. In addition, most existing traffic forecasting graph networks are based on fixed nodes and therefore need more flexibility. Based on the current problem, we propose Dynamic Adaptive Deeper Spatio-Temporal Graph Convolutional Networks (ADSTGCN), a new traffic forecasting model. The model addresses over-smoothing due to network deepening by using dynamic hidden layer connections and adaptively adjusting the hidden layer weights to reduce model degradation. Furthermore, the model can adaptively learn the spatial dependencies in the traffic graph by building the parameter-sharing adaptive matrix, and it can also adaptively adjust the network structure to discover the unknown dynamic changes in the traffic network. We evaluated ADSTGCN using real-world traffic data from the highway and urban road networks, and it shows good performance.

## 1. Introduction

**Figure 1:**
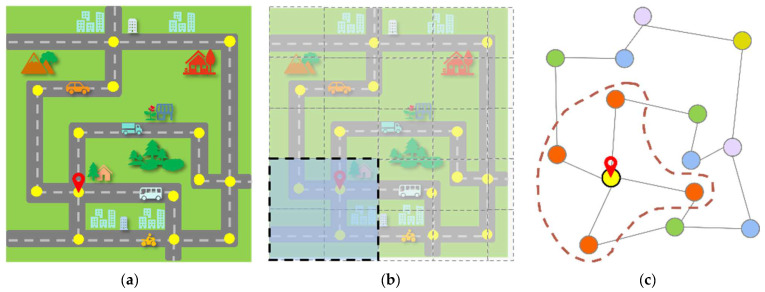
(**a**) Urban road network. (**b**) Description of the regular grid structure of urban road network. (**c**) Description of the irregular graph structure of the urban road network, and different colors represent different neighborhood relationships.

The Intelligent Transportation System (ITS) plays an essential role in urban construction. Reliable and accurate real-time traffic forecasting can help people rationalize travel and ease traffic congestion [1,2]. The development of deep learning has enabled the application of several deep-learning-based forecast models in traffic and transport fields [3,4]. However, traffic conditions have complex, irregular, and nonlinear spatial and temporal relationships [5,6]. The urban road network is complex, irregular, and topological and is challenging to manage conventionally. Graph Convolutional Networks (GCNs) excel in managing non-linear and irregular data, causing them to be extensively applied in traffic forecasting [7,8], as shown in Figure 1. How to construct and optimize graph networks using GCNs to improve traffic forecasting and alleviate traffic congestion is the main problem we address.

**Figure 2:**
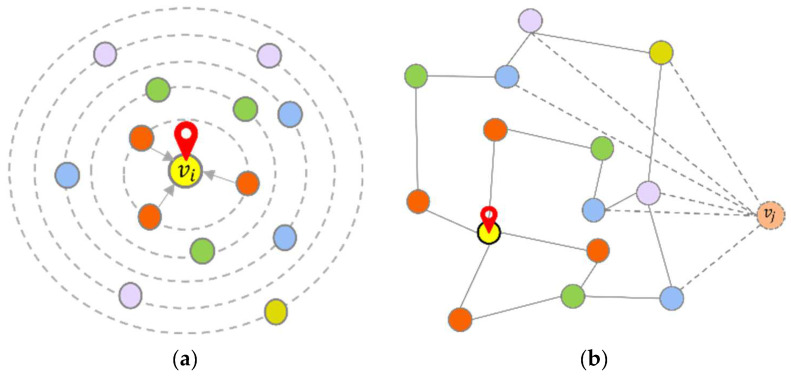
(**a**) The multi-order neighborhood of node $v_{i}$ in the traffic graph. (**b**) For $v_{j}$ nodes that change or are newly added to the graph, the model can adaptively adjust the graph structure and learn its relationship with the surrounding nodes.

The combination of graph convolution and the Gated Recurrent Unit is the first to have improved traffic forecasting [7]. Initially, a purely convolutional approach using graph convolution and 1D Convolution Neural Networks (CNN) was explored in the field of traffic forecasting [8]. They have shown better results in traffic forecasting. However, they are usually shallow networks that aggregate only two- or three-order node neighbor information [7,8,9,10,11]. Deeper models tend to have superior nonlinear expression abilities and extract deeper features [12]. The multi-order neighborhood in the traffic graph is shown in Figure 2a. As the network deepens, the adjacent nodes in the graph structure become increasingly similar, creating an over-smoothing problem [13,14]. This leads to a decrease in forecasting performance. In traffic forecasting studies, skip connections [11,15,16] and GRU architectures [7,17] are used to deepen the overall spatio-temporal model level, but GCN is still a shallow network. Divergent from previous studies, to extract deeper and richer spatial relations in the traffic and increase the node receptive field in the traffic graph, we deepen the neighborhood propagation of the graph network and to mitigate the problem of over-smoothing, and we seek to enhance the connectivity between hidden layers.

The graph construction relies more on the node adjacency matrix. In the traffic forecasting graph, the creation of the adjacency matrix is commonly accomplished by considering the distance, connectivity, or similarity among nodes [7,8,9,10]. These fixed pattern-based graph structures are not the best at discovering unknown hidden spatial relationships between nodes. There are also models that use an adaptive matrix to increase the flexibility of the graph [11,18]. However, they create random matrices that adaptively learn node relationships from the perspective of the feature space, ignoring the composite spatial association information with neighbors and similarity. Different from their work, we propose a parameter-sharing adaptive graph convolution method for traffic forecasting, considering the composite space with near neighbors and similarities and the random feature space in the traffic network. The method discovers unknown dynamic changes in the network by establishing the parameter-sharing adaptive matrix. It can adaptively learn and adjust the spatial dependencies and structures within the traffic according to the changes, as shown in Figure 2b. The main innovative work of this paper is as follows:

1. To address the over-smoothing problem arising from deepening the network layers in multi-step traffic forecasting with Graph Convolutional Networks, we employ a technique of dynamically adjusting hidden layer connections and adaptively modifying the hidden layer weights to prevent model degradation.
2. We propose a parameter-sharing adaptive graph convolution method for multi-step traffic forecasting, which considers the ever-changing complex spatio-temporal relationships within the traffic network. This is able to adaptively learn and adjust the spatial dependencies and structures within the traffic network by building the adaptive matrix for parameter sharing.
3. We propose Dynamic Adaptive Deeper Spatio-Temporal Graph Convolutional Networks (ADSTGCN), a new traffic forecasting model. It uses the diffusion graph convolutional network to obtain spatial dependencies in traffic and the temporal convolutional network to obtain temporal dependencies for better traffic forecasting.
4. We validate our model on two traffic datasets and show better traffic forecasting results than existing advanced baselines.

## 2. Related Work

Multi-step traffic forecasting involves predicting the traffic conditions at various future time intervals from the spatial and temporal dimensions according to the historical traffic conditions in the traffic road network. Its research focuses on the spatio-temporal correlation between the traffic network structure and traffic time series [19]. Recently, deep learning network models have performed outstandingly in traffic forecasting, and their performance is much better than traditional machine learning models [20,21,22]. The spatio-temporal dependence of historical traffic data obtained from sensors can be extracted from the two different dimensions of spatial and temporal, respectively, by using a neural network model.

Extraction of spatial dependencies. GCNs [23,24,25] designed for non-Euclidean data have attracted significant interest in the area of traffic forecasting. According to statistics, most traffic forecasting models since 2019 have used GCNs to model spatial relationships, demonstrating that GCNs research is cutting-edge [1]. The GCNs that are currently available are commonly classified into two categories: spectral domain and spatial domain graph convolution [24,25,26]. The spectral domain graph convolution uses Fourier transform for convolution operations [23]. However, it is very time-consuming to compute the eigenvalue decomposition of the Laplacian matrix, and the model has sizeable parametric complexity. ChebNet utilizes Chebyshev polynomials in the spectral domain as a substitute for the convolution kernel, aiming to decrease the model’s complexity [27]. The GCN simplifies ChebNet by only considering one-order Chebyshev polynomials and only has one parameter per convolution kernel, lowering the model’s complexity [23]. The traffic graph’s spatial representation is extracted using the one-order approximate graph convolution of the Laplacian matrix, which circumvents the spatial neglect issue encountered in recurrent neural networks [8]. Compared with the complex operation of spectral domain graph convolution, spatial graph convolution operates directly on neighborhood nodes, which is more intuitive and flexible. To obtain heterogeneity in the spatial data, after constructing a local spatio-temporal graph, the spatial representation is extracted through spatial graph convolution [3]. Different from the base spatial graph convolution, which only does a linear transformation of its input feature, the diffusion graph convolution takes the aggregation operation on the input feature of its neighbors. A self-attention-mechanism-based information fusion module utilizes diffusion graph convolution to model and comprehend the traffic change relationships of various regions, leveraging the global spatial scope of the entire city [4]. It is also used to model the fusion features extracted from road network graphs and regional graphs [11]. Incorporating diffusion graph convolution, modeling the spatio-temporal dependence between main and auxiliary features is achievable through two segmented spatio-temporal modules [16]. Graph Attention Networks consider the importance of different neighbors and employ the attention mechanism to integrate the information from embedded neighboring nodes. It is used to extract the channel, temporal, and spatial embedding relationships between nodes in the traffic graph [28]. K-hop graph convolution obtains spatial dependences on adjacency matrices constructed using road network connections and competing influence relationships [5]. They have shown better results in traffic forecasting. However, they are usually shallow networks that aggregate only two- or three-order node neighbor information. Skip connections [11,15,16] and GRU architectures [7,17] are used in traffic forecasting studies to deepen the overall spatio-temporal model level also achieve better results, but they are also shallow graph networks. Based on these studies, we focus on intensifying the model hierarchy and deepening the graph network to enlarge the receptive fields of graph nodes, thereby capturing deeper and more intricate spatial relationships within the traffic road network.

The graph structure adjacency matrix determines the GCN performance such that it is one of the main research focuses. Existing studies have generally used the distance between nodes [7,11,29], or the similarity between nodes [9,10,16], to construct adjacency matrices. Other studies have utilized external factors such as POI (Point of Interest) to enhance features along with fusion based on local and global adjacency matrices [3,19,30]. However, these models are based on fixed graph structures and lack the flexibility to capture dynamically changing traffic conditions and road network structures. Some other models use adaptive matrices to increase graph flexibility, learning node feature similarity relationships through two random nodes [11,18]. However, they adaptively learn feature spatial relationships from the feature perspective, and do not adaptively learn the association information from the graph spatial adjacency structure at the same time. On the basis of their work, we adaptively learn and adjust the spatial dependencies and structures within the traffic network by building the adaptive matrix for parameter sharing from the feature and spatial perspectives.

Extraction of temporal dependencies. Traffic forecasting extensively employs recurrent neural networks (RNNs) because of their capacity to memorize and learn both short- and long-term temporal dependencies in sequences [5,7,22,31,32,33]. However, if the dataset is large, the computational load of gating in the RNNs will be large. During rush hour, capturing fluctuations in large traffic volumes is challenging because RNN calculations often rely on the previous step [8]. In certain research works, Convolutional Neural Networks (CNNs) are employed to capture temporal dependencies in traffic forecasting [8,9,17,20,34,35]. However, CNNs perform convolution through input in a window before and after time t, which leads to information leakage after time t. When the historical sequence is long, CNNs need to increase the convolution size to view additional historical information, leading to less efficient training. Thus, Temporal Convolution Networks (TCNs) [36] which combine dilated and causal convolution, have attracted widespread interest in the field of traffic forecasting. TCNs are simple and effective in processing time series data and cannot see future data. Furthermore, TCNs use dilated convolution to obtain a long receptive field with fewer layers, which is beneficial in capturing long-term periodic dependencies. Experimental TCN results have demonstrated that it outperforms RNN in terms of both accuracy and computational time [36]. Temporal dependence at different temporal levels can be obtained by increasing the model temporal receptive field by stacking 1D and 2D causal dilated TCN [10,11,16,29]. In this paper, we use TCN to extract the time dependence of traffic forecasting.

## 3. Methodology

### 3.1. Problem Definition

The primary purpose of multi-step traffic forecasting is to anticipate the traffic conditions for multiple future time steps in the traffic road network, relying on historical traffic data.

**Figure 3:**
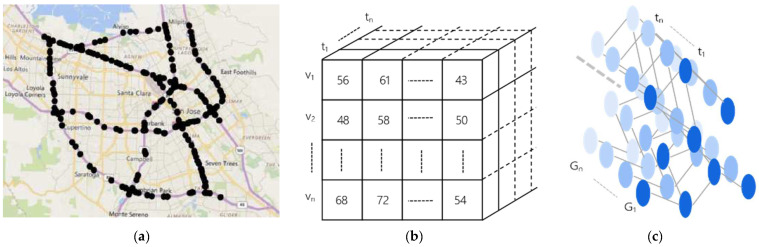
(**a**) Urban highway network. (**b**) The traffic speed of each sensor in the time series. (**c**) Traffic spatio-temporal sequence graph.

**Definition 1.**  *Graph* G*: In this study, the traffic topology is represented by graph* $G\left(V,E\right)$, *as shown in* *Figure 3. The graph’s node set is represented as*$V=\left\{v_{1},v_{2},\ldots \ldots v_{n}\right\}$*. Then, any node* i *can be represented as* $v_{i}$*.* $E=\left\{e_{1},e_{2},\ldots \ldots e_{n}\right\}$*represents the set of connection relationships between all nodes in the graphs.*

**Definition 2.**  *Traffic feature matrix* X*: The traffic conditions of each traffic forecasting sensor are the feature of each node in the graph. In this paper, we mainly study traffic speed as shown in Figure 3b. The traffic speed monitored by all sensors within the road network can be represented by the feature matrix* X, where $X\;\epsilon \;{\mathbb{R}}^{T\times N}$*. The time step is represented by* T*, and the number of nodes is represented by* N*. Then, for any node* i in $G\left(V,E\right)$*, its eigenvalue can be expressed as* $x_{i}$.

**Definition 3.**  *Adjacency matrix* A*: The connectivity among all sensors in the traffic network can be depicted by matrix* A*, commonly referred to as the adjacency matrix,* $A\;\epsilon \;{\mathbb{R}}^{N\times N}$*. In our work, the connectivity of edges in the graph is represented using the distance and similarity between nodes [37].*

**Definition 4.**  *Multi-step traffic forecasting: We slice the time axis into steps every 5 min, denoted by* t*, and the total step is denoted by* T*. In this paper, our objective is to learn a mapping function* f*, which can effectively transform the traffic conditions* X *observed over* P *time steps in the historical data to the predicted traffic conditions* $\hat{Y}$ *over* Q *future time steps. For any node* i*, we can define* ${\hat{Y}}_{i}$ *as:*

(1) $${\hat{Y}}_{i}=f\left(G;x_{i}^{P+1},x_{i}^{P+2},\ldots \ldots ,x_{i}^{P+Q}\right)$$

**Figure 4:**
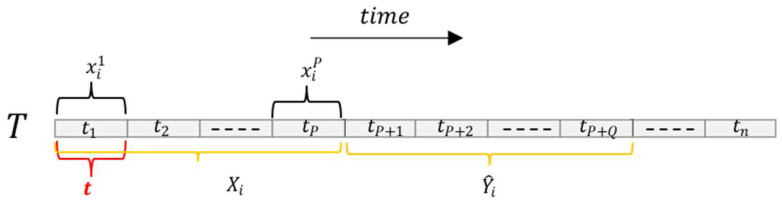
Time series in multi-step traffic forecasting.

*where* P *is the historical time step and* Q *is the predicted time step, as shown in* *Figure 4.*

### 3.2. Overall Architecture

**Figure 5:**
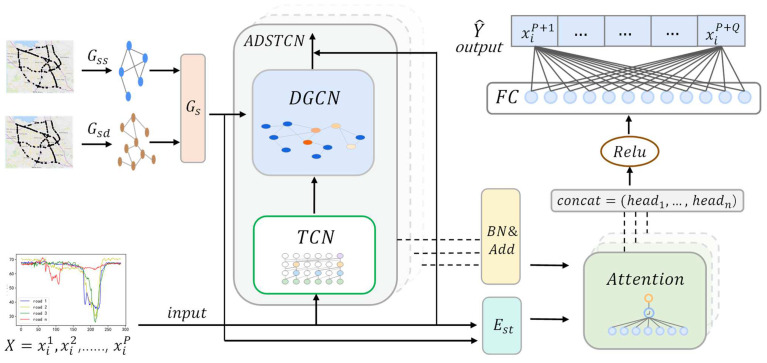
The complete structure of the Dynamic Adaptive Deeper Spatio-Temporal Graph Convolutional Network (ADSTGCN).

Figure 5 shows the overall architecture of the ADSTGCN. The model uses the multi-head attention mechanism [38] to perform multi-strategy fusion transformation on the spatio-temporal dependencies obtained through spatio-temporal convolution and spatio-temporal embedding, respectively. Finally, the forecast results are output after the activation function transformation. In the convolution strategy, TCN convolves the input traffic feature X to obtain the time dependence. Adaptive deep Graph Convolutional Networks obtain spatial dependencies through composite adjacency matrices with distance and similarity relationships. Multiple spatio-temporal layers of the ADSTCN with residual connections [39] are subsequently linked to form the input for the multi-head attention mechanism. In order to further strengthen the spatio-temporal relationship, we integrate the traffic network structure and feature data into $E_{st}$ by embedding and encoding, respectively.

### 3.3. Input Data Processing

Using distances between sensors to create graph adjacencies tends to ignore richer spatial relationships. This paper uses the multi-association graph method in [37] to create graph networks that extract rich spatial dependencies. Spatial static graph $G_{ss}$ represents the neighborhood spatial structure of the traffic network, which is generated based on the distance between road sensors. Spatial dynamic graph $G_{sd}$ is constructed based on the sensors with similar traffic flow in the traffic network with dynamic changes over time. By merging $G_{ss}$ and $G_{sd}$, we create the spatially fused graph $G_{s}$, from which we derive a composite matrix $A_{s}$.

In this paper, we use the One-Hot method to encode time series in traffic data, both daily and weekly, to capture fine-grained adjacent temporal traffic features. According to the dynamic time change, we can identify the time step with a similar traffic flow and obtain the similar function dynamic time step, even if the two time steps are not adjacent. The final temporal dynamic and static features are encoded as $E_{t}$. To further enhance the feature relationship, we utilize the Node2vec method [40] to perform node embedding on the composite adjacency matrix $A_{s}$, resulting in spatial embedding $E_{s}$. Ultimately, we combine the two embeddings to obtain the spatio-temporal embedding. 

### 3.4. Deep Diffusion Graph Convolution

Diffusion-Convolutional Neural Networks assume that information propagates continuously between neighboring nodes according to a certain probability of constant diffusion [24]. Usually, GCN has two operation processes, propagation and transformation. Propagation aggregates each node’s neighborhood information and transforms the aggregated information through a linear transformation or activation function [41,42]. For the feature matrix X, the propagation in the diffusion graph convolutional network can be defined as follows:

(2) $$Z=f\left(W⊙P^{*}X\right)$$

where $Z\;\epsilon \;{\mathbb{R}}^{N\times C}$ denotes the output, $W\;\epsilon \;{\mathbb{R}}^{C\times C}$ denotes the weight matrix, C denotes the number of input and output channels, $P^{*}\in {\mathbb{R}}^{N\times N}$ is the probability transition matrix, and *f* denotes the mapping function. The symbol ⊙ indicates element-wise multiplication. In our work, the matrix $P^{*}$ can be replaced by the composite matrix $A_{s}$. We use the hidden layer output as the input of the next layer, so the new propagation is defined as follows:

(3) $$Z^{0}=X$$

(4) $$Z=\overset{i}{\underset{k=1}{\sum }}W^{k}A_{s}Z^{k-1}\;$$

(5) $$A_{s}=A_{sd}+A_{ss}+I_{N}$$

Here, k refers to the filter, which also signifies the order of the node neighborhood. $Z^{0}$ is the original feature matrix, $A_{s}\in {\mathbb{R}}^{N\times N}$ is the composite adjacency matrix, $A_{sd}$ denotes the static distance matrix, $A_{ss}$ denotes the dynamic similarity matrix, and $I_{N}$ denotes the identity matrix.

If the diffusion order is two in the diffusion graph convolutional network, it means diffusion to the two-order neighbors of the node. For any node $v_{i}$, the propagation of its diffusion convolution is expressed as:

(6) $$Z_{v_{i}}=Z^{0}+Z^{\left(1\right)}+Z^{\left(2\right)}$$

According to the above equations, we define the transformation of diffusion graph convolution as:

(7) $$H=\sigma \left(W^{t}⊙Z\right)=\sigma (W^{t}\left(Z^{0}+Z^{\left(1\right)}+Z^{\left(2\right)}\right)$$

where σ denotes the activation function and H is the final output of the diffusion graph convolution.

In traffic forecasting, shallow GCNs that aggregate two- or three-order neighborhood information can easily lose the deep spatial dependencies of higher-order neighborhoods. However, GCN is prone to over-smoothing with the increase in the aggregated neighborhood order, resulting in the nodes tending to be consistent and indistinguishable, thus reducing the forecasting performance. The core operations of GCNs are propagation and transformation, which significantly impact network performance. It is verified in [42] that decoupling operations on propagation and transformation can expand the node receptive field. Base on this method, on the basis of Equation (3), we decouple the transformations of the features using MLP operations. Then, the new feature matrix $X^{0}$ can be defined as follows:

(8) $$X^{0}=\mathrm{MLP}\left(X\right)$$

(9) $$Z^{0}=X^{0}$$

**Figure 6:**
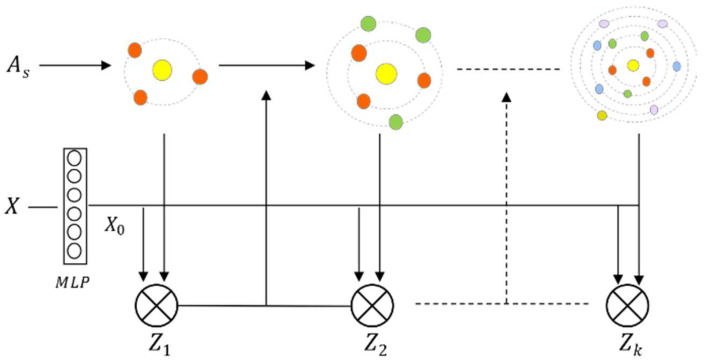
The process of decoupling the feature representation.

The decoupled GCN neighborhood convolution process is shown in Figure 6. Since the deepening of graph networks can suffer from the problem of over-smoothing, to solve this problem, referring to the residual network approach [39], we connect hidden layers to the network, and their weights are adjusted adaptively. The propagation of the deeper graph convolutional can be defined based on Equations (4) and (8) as:

(10) $$X^{\prime }=\left(1-\alpha \right)X+\alpha X^{0}+\beta \left(X+X^{0}\right)$$

(11) $$Z=\overset{i}{\underset{k=1}{\sum }}W^{k}A_{s}X^{\prime }$$

where α and β are hyperparameters, α belongs to the range $\left(0,1\right)$, and β is equal to $1-k^{-1}$. Here, k represents the node convolution order. The parameter β increases as k grows, and this helps to mitigate model degradation.

### 3.5. Adaptive Deep Graph Convolution

Although composite adjacency matrices based on node distance and similarity function can simultaneously capture the spatial relationship between adjacent and non-adjacent nodes, they are built based on a fixed structure and are not ideal for discovering the unknown hidden spatial relations between nodes. Traffic flow can change in a complex way depending on various external factors, and a fixed graph structure makes it difficult to extract more information from the challenging changes. We create an adaptive matrix to improve the flexibility of the graph. It can acquire the dependencies in different spaces through parameter sharing and adaptively learns the unknown changing relationships in the network. We set two randomly initialized matrices, fuse them and use a nonlinear activation function to activate, so that the adaptive matrix is defined as follows:

(12) $$A_{adp}=\sigma \left(A_{1}A_{2}\right)$$

where $A_{adp}$ is the adaptation matrix, σ is the activation function, and $A_{1},A_{2}\;\epsilon \;{\mathbb{R}}^{N\times N}$ are two random initialization matrices representing random sensor nodes in the traffic network. According to the above equation, the propagation of adaptive graph convolution can be defined as:

(13) $$Z_{adp}=W^{a}\sigma \left(A_{1}A_{2}\right)X$$

Adaptive adjacency matrices feature spaces with randomness, and composite adjacency matrices are spaces possessing proximity and similarity. They have some common features, although their parameters are different. By adopting parameter sharing, we extract common features to further strengthen the fusion of spatial and feature information. We can define the spatial graph convolution and adaptive graph convolution with the same shared weights as:

(14) $$Z_{sp}=W^{c}A_{s}X$$

(15) $$Z_{adp}{}^{\prime }=W^{c}A_{adp}X$$

where $Z_{sp}$ denotes spatial graph convolution, $Z_{adp}$ denotes adaptive graph convolution, and $W^{c}\;\epsilon \;{\mathbb{R}}^{C\times C}$ is the shared weight matrix. Then, the shared graph convolution can be defined as:

(16) $$Z_{com}=\left(Z_{sp}+Z_{adp}{}^{\prime }\right)/2$$

According to Equations (13) and (16), we can define the propagation of the parameter-sharing adaptive graph convolution as:

(17) $$Z_{adp\_c}=Z_{adp}+Z_{com}$$

**Figure 7:**
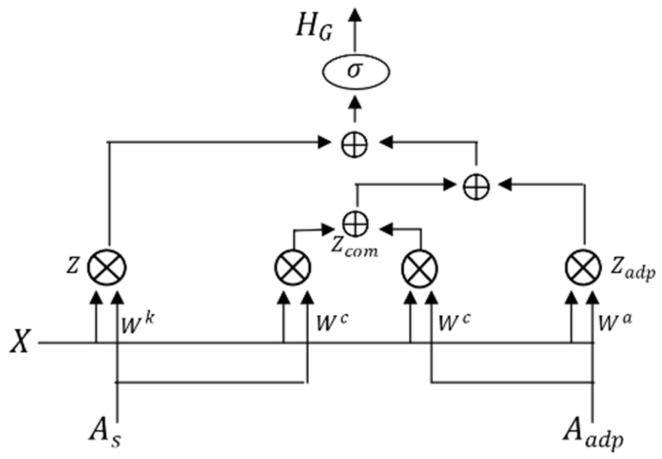
Adaptive graph convolution with parameter sharing.

According to Equations (11) and (17), after transformation, as shown in Figure 7, we finally define the adaptive deeper graph convolution as:

(18) $$H_{G}=\sigma \left(\sum_{k=1}^{i}W^{k}A_{s}X^{\prime }+Z_{adp\_c}\right)$$

### 3.6. Dilated Causal Temporal Convolution

A Temporal Convolution Network (TCN) [36] is widely used in time series research because the inability to see future data during propagation avoids information leakage. It employs dilated convolution to enlarge the receptive field, enabling the capture of longer temporal relationships. In this study, we use a TCN to capture temporal relationships in the traffic flow. It can be defined as:

(19) $$H^{\prime }=\sum_{i=0}^{k^{\prime }-1}f\cdot X_{s-d\cdot i}$$

where f is the 1-D filter, s is any time step within the set T, d is the dilation factor, and $k^{\prime }$ is the kernel size. In this paper, we set $k^{\prime }$ = 2, that is, the time convolution on the s-th time step involves convolving the upper layer’s time step with the (s−d)-th time step, then the above equation can be simplified as:

(20) $$H^{\prime }=f\cdot X_{s}+f\cdot X_{s-d}$$

**Figure 8:**
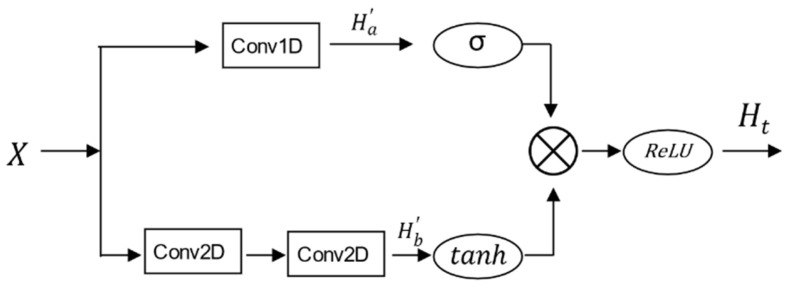
TCN gating mechanism.

To further extract richer time dependencies, we add a gating mechanism:

(21) $$H_{T}=ReLU\left(sigmoid\left(H_{a}^{\prime }\right)*\tanh\left(H_{b}^{\prime }\right)\right)$$

where $H_{a}^{\prime }$ denotes the 1D temporal convolution operation in the temporal dimension and $H_{b}^{\prime }$ denotes the 2D temporal convolution operation in both the spatial and temporal dimensions. The sigmoid activation function filters weaken relations in the 1D convolution, and the tanh activation function controls the 2D convolution result between (−1, 1). Both activation functions are multiplied to highlight the important information, and the ReLU activation function is used to eliminate weak connections in the TCN to obtain the final temporal dependencies. We use double-layer convolution in 2D temporal convolution in both spatial and temporal dimensions to capture additional spatio-temporal relationships, as shown in Figure 8.

### 3.7. Attention Mechanism

To strengthen the spatio-temporal dependency extraction, we combine the spatio-temporal embedding $E_{st}$ with the spatio-temporal convolutional layer output to perform multi-strategy fusion transformation through the multi-head attention to obtain the forecast result. In this study, we divide the space-time embedding $E_{st}$ into historical spatio-temporal embedding $E_{st\_h}$ and predictive spatio-temporal embedding $E_{st\_p}$ and acquire the importance weight of the embedding predicted from historical embedding. Referring to the attention mechanism, we define single-head attention as:

(22) $$H^{\prime }=\sum_{i=1}^{n}\alpha_{st}\cdot V$$

(23) $$\alpha_{st}=softmax\left(\left(E_{st\_p}\cdot E_{st\_h}{}^{T}\right)\cdot h^{-0.5}\right)\;$$

where $\alpha_{st}$ denotes the importance coefficient of spatio-temporal attention, V denotes the spatio-temporal dependency obtained after stacking ADSTCN layers, $H^{\prime }$ denotes the output result of single-head attention, softmax is the activation function, and h is the quantity of attention heads.

We concatenate the multi-head attention output to obtain the fusion output result and transform the attention mechanism, which will be converted by the activation function and fully connected layer into the final forecast result. According to Equation (10), the output result after fusion and the multi-head attention mechanism transformation is defined as:

(24) $$H_{att}=concat\left(H_{1},H_{2},\ldots \ldots ,H_{h}\right)$$

## 4. Experiments

In this section, we assess the performance of the ADSTGCN model using two real datasets, namely the highway network and the urban road network. We compare and analyze our model’s experimental outcomes against nine traffic forecasting baseline models to validate its effectiveness. Additionally, we conduct ablation studies and analyze the pivotal components in the model.

### 4.1. DataSets

**Figure 9:**
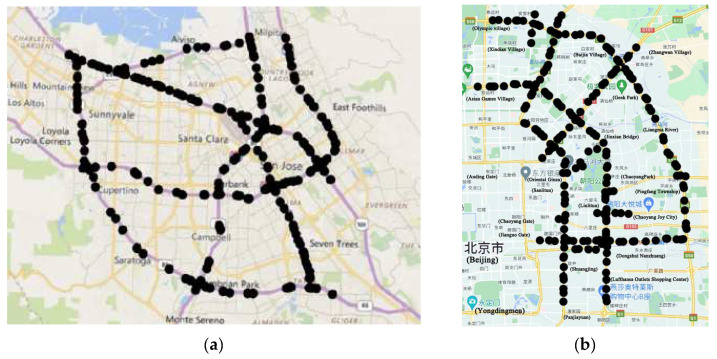
(**a**) The PEMS_BAY dataset’s sensor distribution. (**b**) The NE_BJ dataset’s sensor distribution.

In our experiment, we select two real traffic datasets, as shown in Figure 9. One is the highway network dataset PEMS_BAY. The CalTrans Performance Measurement System collects it and has 325 sensors. It collected data for six months, from 1 January 2017 to 31 May 2017. The traffic speed is high, and the traffic situation is comparatively simple as PEMS_BAY involves high-speed road network data. Another dataset used in this study is the NE_BJ road network dataset, comprising 500 sensors, and collected through Navigation data in Northeast Beijing for a duration of one month. It spans between 1 July 2020 and 31 July 2020. The NE_BJ dataset is the real dataset of the main roads within the Beijing urban area. It is more complex and congested than freeway traffic, making it more challenging to forecast traffic. It also has more research value.

Traffic flow data is collected every 30 s, and the unit of speed is km/h. Before the experiment, the collected data were pre-processed and aggregated into 5 min time steps, with one hour of 12 time steps. All data are arranged into time series according to the time step, which is then used as the model’s input data. The data is separated into three parts, with proportions of 7:2:1 for the training, test, and validation sets.

### 4.2. Experimental Settings

We conduct experiments using PyTorch 1.10 on a GeForce RTX 2080Ti GPU. The learning rate is 1 × 10^−3^, and the batch size is 16. The order of neighborhood is 8, and the kernel size of the TCN is 2. The time step T is configured to be 12. We use MAE, RMSE, and MAP to evaluate the performance of the models, which are often used in traffic forecasting model evaluation.

### 4.3. Baselines

During the experiments, we conducted a comparison between ADSTGCN and nine baseline methods. HA [43]: The forecast result is the average of all historical records. VAR [44]: The real-time fluctuation of traffic state can be obtained, and is frequently employed in multivariate time series models. FC-LSTM [45]: A recurrent neural network with LSTM hidden units is fully connected. DCRNN [7]: Graph convolutions are embedded into GRU, and modeled with encoder–decoder architecture for traffic forecasting. STGCN [8]: Spatio-temporal relationships are modelled using pure convolutions to predict traffic with fewer parameters and faster training. GWnet [11]: The use of diffusion graph convolution and an adaptive matrix to obtain better short-term forecast effects. AGCRN [18]: The adjacency matrix is obtained by data-adaptive learning of intrinsic hidden associations between nodes. GMAN [21]: The spatio-temporal representation is extracted according to the random walk of graph nodes and the attention mechanism, and the encoder–decoder architecture is used to model and improve poor medium- and long-term traffic forecasts. MTGNN [46]: Multivariate time series are processed with or without predefined graph structures through a joint framework for modeling learning graph and time series data.

### 4.4. Experimental Results

We compare the ADSTGCN with the baseline on two real datasets, PEMS_BAY and NE_BJ. The forecasts for each model for the next 15 min, 30 min, and 60 min are presented in Table 1, and all models are evaluated using the MAE, RMSE, and MAPE metrics.

According to the results presented in Table 1, the non-neural network models, HA and VAR, perform poorly in traffic forecasting, and their learning ability for features is not as strong as that of the neural network models. Conversely, the neural network models achieve better performance in the forecast. After conducting a comprehensive comparison of the two datasets, it is observed that the ADSTGCN model’s enhancement of the graph network results in superior performance compared to other baseline models in terms of MAE, RMSE, and MAPE. Through the deepening of the GCN, the ADSTGCN is capable of extracting more profound and intricate spatial relationships, leading to improved long-term forecasting performance, particularly in the Beijing inner city roads with more complex traffic conditions. Additionally, ADSTGCN incorporates an adaptive matrix for parameter sharing, enhancing the flexibility of the graph convolutional network model and facilitating the capture of evolving traffic states, resulting in improved performance.

On the PEMS_BAY dataset, the ADSTGCN model exhibits superior forecast performance for both short-term (15 min) and long-term (60 min) forecasts. GMAN model uses RNN to achieve better long-term forecast results, and ADSTGCN outperforms it in short-term forecasts by 4.48% in MAE. For long-term forecasting results, both models exhibit a similar performance. GWnet achieves superior short-term forecasting results using a purely convolutional model, and ADSTGCN outperforms it by 1.54% in MAE for short-term forecasts and by 4.62% in MAE for long-term forecasts. MTGNN improves the extraction of spatio-temporal dependencies using hybrid jump propagation and achieves a better comprehensive result in both short-term and long-term forecasts. ADSTGCN improves short-term and long-term forecasts compared to it, where short-term forecasts outperform it by 3.03% in MAE, and long-term forecasts outperform it by 4.12% in MAE.

ADSTGCN shows better forecast results in both short-term and long-term forecasts of NE_BJ datasets under more complex traffic situations, with better long-term forecast results. GMAN uses RNN to achieve better long-term forecast results, and ADSTGCN outperforms it by 1.74% in MAE for long-term forecasts and by 7.35% in MAE for short-term forecasts. ADSTGCN’s short-term forecast is worse than GWnet in MAE, and its MAE is 1.07% behind GWnet’s, but its long-term forecast is 5.21% better than GWnet in MAE. ADSTGCN is significantly affected by external factors in more complex traffic situations in the short term, and the forecast effect is insufficient. Still, ADSTGCN has a more stable performance in medium- and long-term forecasts.

**Figure 10:**
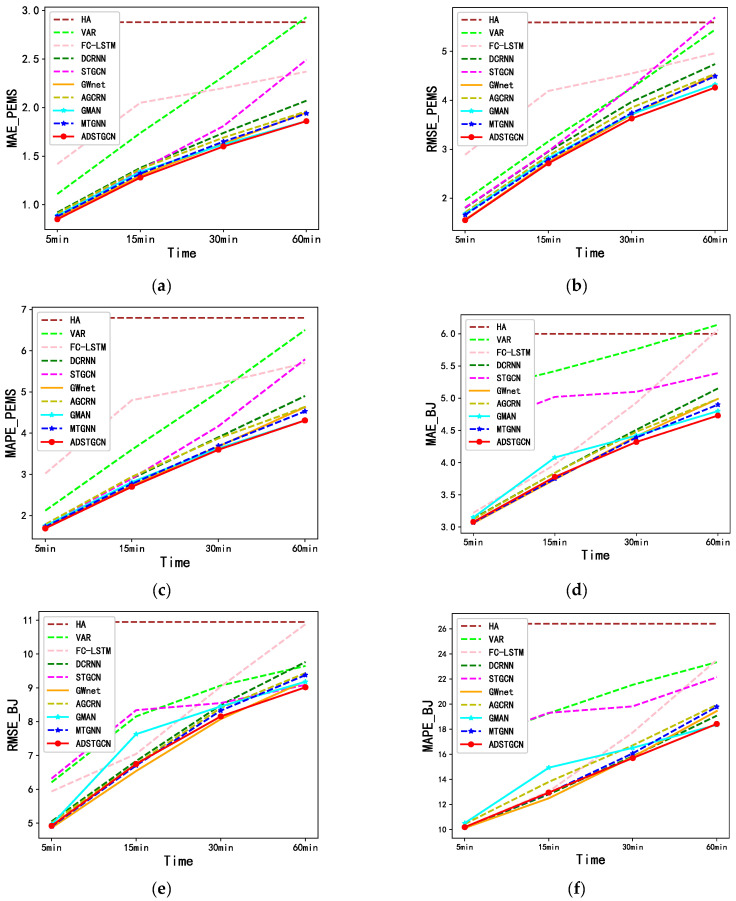
Performance comparison of ADSTGCN with each baseline model. (**a**) MAE(PEMS_BAY); (**b**) RMSE(PEMS_BAY); (**c**) MAPE(PEMS_BAY); (**d**) MAE(NE_BJ); (**e**) RMSE(NE_BJ); (**f**) MAPE(NE_BJ).

DCRNN and AGCRN use GCN and RNN to model spatio-temporal relationships, as RNNs are good at sequence data and have better long-term forecast performance than short-term. STGCN, GWnet, and MTGNN use GCN and CNN to model spatio-temporal relationships, are more concise, and achieve better short-term forecast results than long-term. The GMAN model adopts the multi-attention model and an encoding–decoding mechanism to achieve better long-term forecasts than other baseline models. On the basis of GCN, ADSTGCN acquires deeper spatial neighborhood dependencies, extracts richer shared features, and uses adaptive matrices to make the network more flexible. This enables the extraction of richer traffic graph features and learning of more flexible traffic graph structures, and therefore the model improves the forecasting performance. Deepening the graph network makes it easier to discover deeper and more complex spatial relationships between neighboring nodes, thus achieving better performance in long-term forecasting. Figure 10 compares the forecasting performance of ADSTGCN and the nine baseline models on the PEMS_BAY and NE_BJ datasets, respectively.

**Figure 11:**
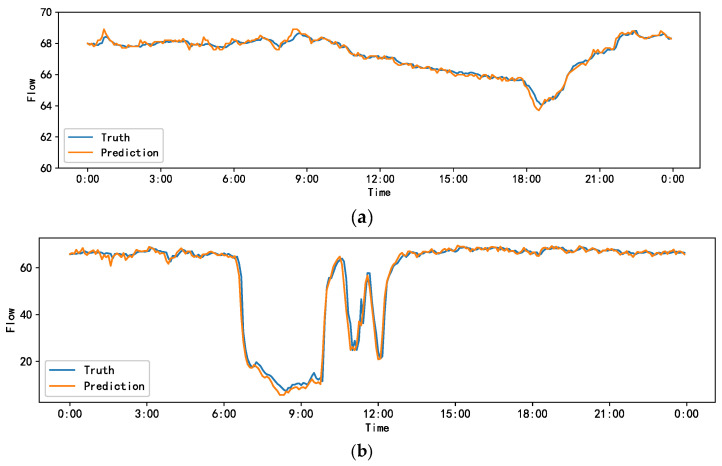
Comparison of the truth and predicted values of the ADSTGCN on the PEMS_BAY and NE_BJ datasets. (**a**) PEMS_BAY; (**b**) NE_BJ.

Figure 11 compares the actual and predicted traffic forecasting of the ADSTGCN on the PEMS_BAY and NE_BJ datasets on a specific day.

### 4.5. Ablation Study

**Figure 12:**
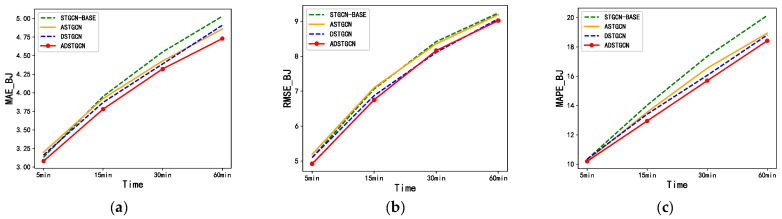
Performance comparison of ADSTGCN with each ablation model in the NE_BJ dataset. (**a**) MAE(NE_BJ); (**b**) RMSE(NE_BJ); (**c**) MAPE(NE_BJ).

In this section, we conduct experimental ablation research on key model components to verify the method’s effectiveness and help us to improve the model further. We study the following ablation models: STGCN: a base model that only includes a two-order neighborhood GCN; DSTGCN: an STGCN-based model that deepens GCN neighborhoods; ASTGCN: a model that adds a parameter-sharing adaptive adjacency matrix to the STGCN. Our proposed ADSTGCN deepens the GCN neighborhood based on the STGCN and adds a parameter-sharing adaptive adjacency matrix model. Taking the NE_BJ dataset as an example, we compare the MAE, RMSE, and MAPE values of the ablation and ADSTGCN model forecast results at 15, 30, and 60 min, respectively, as shown in Figure 12.

The figure shows that the NE_BJ dataset, which has more complex traffic situations, exhibits favorable short-term and long-term traffic forecasting performance when using the ADSTGCN model with the parameter-sharing adaptive adjacency matrix and the adaptive hidden layer connection method. The overall performance of the ASTGCN model using the parameter-sharing adaptive adjacency matrix is better than the basic STGCN model, and its long-term forecast effect is better than its short-term forecast. The comprehensive performance of the DSTGCN using the adaptive hidden layer connection method is better than that of the basic STGCN model. Because this method can deepen the model and restrain the over-smoothing problem, the short-term and long-term forecast performance is relatively stable.

**Figure 13:**
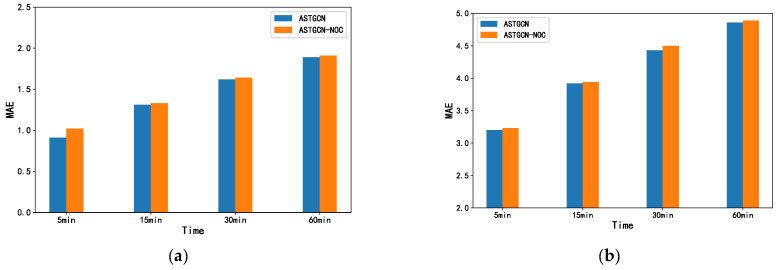
Comparison of the impact of parameter sharing on the forecast performance of the adaptive adjacency matrix on the PEMS_BAY and NE_BJ datasets. (**a**) MAE(PEMS_BAY); (**b**) MAE(NE_BJ).

We compare the ASTGCN with the ASTGCN-NOC adaptive matrix with the parameter sharing removed on the PEMS-BAY and NE_BJ datasets to verify the superior effect of parameter sharing on adaptive matrix adjacency. Their contrasting results on MAE values are shown in Figure 13. It can be seen from the figure that using the parameter-sharing method to extract the adjacent composite and random-feature-space common features further influence the model forecast effect. Adjacent composite spatial convolution is based on composite spatial matrices with neighbors and similarities, while random eigenspace convolution is based on adaptive and eigenspace matrices. In addition to their different parameters, they also have something in common. By extracting the common features of feature and space, the fusion of feature and space is further strengthened to improve the forecast effect.

## 5. Conclusions

This paper mainly studies the traffic flow forecasting problem using deep Graph Convolutional Networks, as well as traffic road network graph adaptability, and the use of multi-strategy information extraction in traffic forecasting models. We introduce a novel traffic forecasting model, Dynamic Adaptive Deeper Spatio-Temporal Graph Convolutional Networks for Multi-Step Traffic Forecasting (ADSTGCN), using GCN and TCN to obtain spatio-temporal relationships, respectively. The model deepens the neighborhood convolution of the graph while mitigating the network over-smoothing problem using hidden layer connectivity, allowing the model to extract deeper and richer features. The flexibility of node structures in traffic graphs is enhanced using a parameter-sharing adaptive approach. The ADSTGCN performs well when evaluated on two real datasets, highways and urban roads. In our future research, we aim to optimize the model further, validate the model on more comprehensive experimental environments and datasets, and improve the model’s efficiency.

## Figures and Tables

**Table 1:** Evaluation of traffic forecasting performance of various models on PEMS_BAY and NE_BJ datasets.

	Method	15 min			30 min			60 min		
		MAE	RMSE	MAPE	MAE	RMSE	MAPE	MAE	RMSE	MAPE
PEMS_BAY	HA [43]	2.88	5.59	6.80%	2.88	5.59	6.80%	2.88	5.59	6.80%
	VAR [44]	1.74	3.16	3.60%	2.32	4.25	5.00%	2.93	5.44	6.50%
	FC-LSTM [45]	2.05	4.19	4.80%	2.20	4.55	5.20%	2.37	4.96	5.70%
	DCRNN [7]	1.38	2.95	2.90%	1.74	3.97	3.90%	2.07	4.74	4.90%
	STGCN [8]	1.36	2.96	2.90%	1.81	4.27	4.17%	2.49	5.69	5.79%
	GWnet [11]	1.30	2.74	2.73%	1.63	3.70	3.67%	1.95	4.52	4.63%
	AGCRN [18]	1.37	2.87	2.94%	1.69	3.85	3.87%	1.96	4.54	4.64%
	GMAN [21]	1.34	2.82	2.81%	1.62	3.72	3.63%	1.86	4.32	4.31%
	MTGNN [46]	1.32	2.79	2.77%	1.65	3.74	3.69%	1.94	4.49	4.53%
	ADSTGCN	1.28	2.71	2.70%	1.60	3.63	3.60%	1.86	4.26	4.31%
NE_BJ	HA [43]	6.00	10.95	26.40%	6.00	10.95	26.40%	6.00	10.95	26.40%
	VAR [44]	5.42	8.16	19.28%	5.76	9.07	21.53%	6.14	9.65	23.33%
	FC-LSTM [45]	3.97	7.05	13.05%	4.93	9.04	17.74%	6.06	10.88	23.52%
	DCRNN [7]	3.84	6.84	12.82%	4.51	8.49	15.84%	5.15	9.77	19.08%
	STGCN [8]	5.02	8.34	19.31%	5.10	8.55	19.82%	5.39	9.09	22.14%
	GWnet [11]	3.74	6.54	12.49%	4.41	8.08	15.79%	4.99	9.20	19.45%
	AGCRN [18]	3.84	6.75	13.80%	4.48	8.41	16.70%	4.99	9.44	19.94%
	GMAN [21]	4.08	7.63	14.94%	4.42	8.45	16.51%	4.80	9.18	18.36%
	MTGNN [46]	3.75	6.71	12.91%	4.39	8.33	16.07%	4.90	9.38	19.79%
	ADSTGCN	3.78	6.75	12.95%	4.32	8.16	15.70%	4.73	9.02	18.42%

## Data Availability

The data presented in this study are public datasets that can be downloaded from the public data provider https://pems.dot.ca.gov (accessed on 2 August 2023).
